# Intervention to Increase Uptake of Recommended Vaccines in an Inner-City HIV Care Clinic

**DOI:** 10.1007/s10461-025-04948-9

**Published:** 2025-11-12

**Authors:** Jose G. Castro, Valeria Botero, Allan Rodriguez

**Affiliations:** Department of Medicine – Infectious Diseases, University of Miami Miller School of Medicine, 1120 NW 14th Street, Suite 856, Miami, FL USA

**Keywords:** HIV care, Vaccination rates, Intervention strategies, Immunization practices, Urban health services, Atención del VIH, Tasas de vacunación, Estrategias de intervención, Prácticas de inmunización, Servicios de salud urbana

## Abstract

This study investigates the effectiveness of a targeted intervention aimed at improving vaccination rates among People Living with HIV (PLWH) at the University of Miami/Jackson Memorial Hospital’s Adult HIV Outpatient Clinic, a key provider for a predominantly minority population in Miami-Dade County, Florida. Despite the established efficacy of vaccines and their endorsement by various health agencies, vaccination rates among PLWH remain suboptimal, particularly in minority groups. Our intervention, structured around the CERPS framework (Champion, Education, Reminder, Performance feedback, and Standing orders), was designed to address these disparities by increasing educational outreach, reminders, and accessibility to vaccinations within the clinic. The study was conducted over two years and included 400 Hispanic participants, randomized into intervention and control groups. Results showed high retention and vaccination rates that approached or exceeded national averages. However, comparisons between the intervention and control groups were not significant, suggesting potential benefits from increased general awareness and inadvertent cross-group contamination. The findings highlight the complexities of measuring intervention impacts in real-world settings and underscore the need for clinic-wide strategies to enhance vaccination rates. This study adds to the understanding of effective strategies to increase vaccination uptake in urban HIV care settings and highlights the need for further research into provider and patient-centered barriers and facilitators.

## Resumen

Este estudio investiga la efectividad de una intervención dirigida a mejorar las tasas de vacunación entre las Personas Viviendo con VIH (PVVH) en la Clínica de VIH para Adultos del Hospital Universitario de Miami/Jackson Memorial, un proveedor clave para una población predominantemente minoritaria en el Condado de Miami-Dade, Florida. A pesar de la eficacia establecida de las vacunas y su respaldo por diversas agencias de salud, las tasas de vacunación entre las PVVH siguen siendo subóptimas, especialmente en los grupos minoritarios. Nuestra intervención, estructurada alrededor del marco CERPS (Promotor, Educación, Recordatorios, Retroalimentación de desempeño y Órdenes permanentes), fue diseñada para abordar estas disparidades aumentando la divulgación educativa, los recordatorios y la accesibilidad a las vacunas dentro de la clínica. El estudio se llevó a cabo durante dos años e incluyó 400 participantes hispanos, aleatorizados en grupos de intervención y control. Los resultados indicaron altas tasas de retención y un aumento en las tasas de vacunación que se acercaron o superaron los promedios nacionales. Sin embargo, las comparaciones entre los grupos de intervención y control no mostraron diferencias significativas, lo que sugiere beneficios potenciales de un mayor conocimiento general y contaminación cruzada inadvertida entre grupos. Los hallazgos destacan las complejidades de medir los impactos de las intervenciones en entornos del mundo real y subrayan la necesidad de estrategias a nivel local de la clínica para mejorar las tasas de vacunación. Este estudio contribuye a la comprensión de estrategias efectivas para aumentar la aceptación de vacunas en entornos de atención de salud urbana y sugiere más investigaciones sobre las barreras y facilitadores centrados en el proveedor y el paciente.

## Introduction

Vaccines are among the most effective interventions in modern medicine [1] and are also among the most cost-effective public health interventions to protect people from infectious diseases and protect against devastating epidemics. Vaccines also benefit society by preventing hospitalizations, avoiding long-term disability, and reducing absence from work [1]. Due to its importance as a preventive health strategy, diverse agencies commonly choose to increase vaccination coverage as a measure of medical service quality control. For example, two of the eleven indicators of the Core Clinical Performance created by the U.S. Health Resources and Services Administration (HRSA) HIV/AIDS Bureau (HAB) for Adults and Adolescents specifically target vaccination rates including influenza vaccination and pneumococcal vaccination [2]. Although these services are covered in the United States by Medicare, Medicaid, and many private insurance plans under the Affordable Care Act, millions of children, adolescents, and adults still go without this preventive services [3]. A recent report from the CDC stated that despite recommendations for vaccination throughout life to prevent vaccine-preventable diseases and their sequelae; adult vaccination coverage remains low for most routinely recommended vaccines [4]. Lower vaccination is even more concerning in disadvantaged populations as has been documented that significant racial and ethnic disparities exist in adult immunization rates, even when socioeconomic status, access to care, and insurance coverage are taken in consideration. Immunization coverage among White adults is 67%, compared to rates about 20% points lower among Black and Hispanic adults [5].

Vaccine protective efficacy is of greater necessity in People living with HIV (PLWH), because people with HIV infection often have an increased risk of infection or experience more severe morbidity following exposure to vaccine-preventable diseases [6–11]. However, vaccination rates are frequently low in this population [11]. Providing appropriate immunizations is a well-accepted and important component of comprehensive HIV clinical care. The Advisory Committee on Immunization Practices (ACIP) provides annual recommendations for routine immunizations of adults, including specific recommendations for PLWH [12]. In addition, the US Guidelines for the Prevention and Treatment of Opportunistic Infections also provides specific vaccine recommendations for PLWH [13]. Because of the importance of increasing coverage of recommended vaccinations, several strategies have been designed and proven effective. A comprehensive review of the literature has identified the following interventions as “effective or highly effective” [14]: Client Reminder/Recall, multicomponent Interventions that include Education, reducing out of pockets costs and provider reminder/recall. These strategies have not been specifically validated in either racial and ethnic minorities or in clinics serving PLWH. The University of Miami/Jackson Memorial Hospital UM/JMH Adult HIV vaccination rates are lower than national averages. Despite the high level of expertise and interdisciplinary approach to primary care for PLWH in this clinic, there still are areas of care that require improvement when compared to national averages, specifically for immunizations. At UM/JMMC, influenza vaccination coverage was 46%, compared to the U.S. average of 65%. Pneumococcal vaccination coverage was 36%, below the national average of 70%. Considering this shortcoming and to address the lower rates of immunizations among racial and ethnic minorities, we designed an intervention in English and Spanish to address barriers that hinder immunization rates such as navigating insurance coverage, low education, low retention in care, being MSM or having a low CD4 to try to improve immunization rates [11]. Its aim was to increase the uptake of vaccinations among PLW.H, particularly Hispanics. The objective of this study was to adapt, implement, and evaluate a validated intervention designed to increase vaccination a through patient education and reminders, standing orders for vaccinations in the clinic and giving information to Providers their patients’ vaccination rates.

## Methodology

To evaluate the intervention’s effectiveness, we measured vaccination rates for influenza and pneumococcal among Hispanics lining with HIV in 2015 and 2016 and compared them with clinic baseline rates. This strategy facilitated comparison with other cohorts of one-time vaccinations during the study period.

### Ethics Statement

Permission was granted by the University of Miami Institutional Review Board and the Jackson Memorial Hospital Clinical Research Review Committee.

#### Setting

The University of Miami/MH (UM/JMH) Adult HIV Outpatient Clinic provides HIV care to approximately 3000 distinct patients annually. During the study year, 2600 unique patients were seen at least once, reflecting a high proportion of irregular attendees. It is the largest single-location HIV clinic provider in Florida and a primary entry point for HIV care in Miami-Dade County. Most of the patients are from racial/ethnic minority group (57.9% Black; 36.4% Hispanic/Latino); 49% are foreign born, and 38% are women. Uninsured individuals (Ryan White or no insurance) represent 40% of the clinic population, followed by Medicaid recipients (35%). Overall, 54% of patients have an AIDS diagnosis; among Hispanics, the rate is 47%. The clinic reflects the regional realities of HIV clinics in the Southeast U.S., where the median number of patients seen in clinics is higher, the average wait times for appointments are higher, and the highest ratios of patients to clinicians have been reported relative to the rest of the U.S [15]. Most patients are of a lower socioeconomic status and require substantial assistance in accessing medical care.

The UM/JMH Adult HIV Outpatient Clinic is staffed by University of Miami faculty along with members of an interdisciplinary treatment team from both Jacskon Memorial Medical Center (JMMC) and UM. Patient care is provided by 12 attending physicians (Department of Infectious Diseases, University of Miami Miller School of Medicine), one advanced practice registered nurse and one physician assistant.

#### Study Intervention

The intervention was based on the CERPS framework (Champion, Education, Reminder, Performance feedback and Standing orders), which has been shown to improve immunization rates and was successfully applied in the Veterans Health Administration [16] It includes: (1) champions for adult immunization (Peer Navigator) [17–20], (2) education for providers, patients, families and caregivers [21–25], (3) reminders to vaccinate, (4) performance feedback for providers [26–30], and standing orders for adult immunization [31].

The CERPS intervention aimed to achieve ≥ 90% immunization coverage and to ensure vaccine administration, follow-up, and documentation were sustained. The intervention was delivered by study team members.

Intervention components and delivery:

1. Education (Study Coordinator): Patients in the intervention group received a 30-minute education session, individually or in groups, covering vaccine indications and contraindications in HIV, as well as benefits and potential side effects.
2. Reminders (Peer Navigator and Study Coordinator): Intervention group patients received vaccination reminders, and providers were given annotated daily clinic schedules identifying their intervention patients needing vaccination.
3. Performance Feedback (PI): The PI provided providers with quarterly reports on their patients’ vaccination rates.
4. Standing orders (PI, Clinic Director): Standing orders for routine vaccinations were incorporated into the electronic medical record for intervention group patients.

Control group patients received standard HIV education classes held twice monthly, with no other elements of the CREPS intervention. Classes, typically offered in Spanish, covered HIV basics including transmission, disease course, medication adherence and side effects, safer sex practices, HIV status disclosure, and prevention of opportunistic infections.

#### Study Participants

Between February 2016 to February 2018, participants were recruited by study staff during regularly scheduled care appointments. Eligible participants over the age of 18, had documented HIV infection, and self-identified as Hispanic. Exclusion criteria included allergy to vaccine components. Participants received a $40 honorarium for participation. All study materials (flyers, consent forms, surveys) were available in English and Spanish. All study staff were bilingual, and interactions were conducted in the patient’s preferred language.

After providing consent, participants completed questionnaires on demographics and factors that might influence vaccination. Immunization records were reviewed to determine the need for influenza and pneumococcal vaccination. During the study period, clinic standard was to administer the 23-valent polysaccharide vaccine (PPSV23) every five years. All randomized participants were eligible for influenza vaccination, which is given annually. Need for pneumococcal vaccine was determined based on prior PPSV23 within five years. PCV13 was indicated if no dose had been given in the past year following a previous PPSV23. After baseline evaluation, participants were randomized to the intervention group (IG, *n* = 200) or the standard of care group (SOC, *n* = 200). Randomization occurred at baseline, and participants remained in the same group throughout; They were assessed twice: once in year 1 and again in year 2. Vaccination rates were recorded at the end of each year.

#### Evaluation

To evaluate the effectiveness, we collected both objective and subjective health information, including influenza and pneumococcal vaccination rates in 2015 and 2016, and compare them to clinic baseline rates. Pneumococcal vaccination rates were measured as overall cohort coverage per year rather than by individual eligibility. This approach facilitated comparison with other cohorts. Participants were assessed on demographics (ethnicity, marital status, living arrangement, religion, education, employment, income), healthcare information (insurance status, preferred services), and known factors influencing vaccination adherence (lack of insurance, low education, financial stressors, missed appointments) [11]. Additional measures included self-reported health status, health literacy (Test of Functional Health Literacy in Adults), medication adherence (IMB Medication Adherence Questionnaire), HIV knowledge (HIV Knowledge Questionnaire), quality of life (MOS-HIV Health Survey, MOS-HRQoL), and acculturation (Bidimensional Acculturation Scale for Hispanics). Data were collected at two time points to assess whether changes in these factors influenced vaccination rates beyond baseline. Surveys were available in English and Spanish.

For individual predictors, ANOVA was used for continuous variables and chi-square tests for categorical outcomes to compare intervention and control groups. For ANOVA, equality of variances was tested using the Brown–Forsythe statistic; when violated, the Welch’s F-test was applied. Bivariate models were fit first; variables with p values ≤ 0.1 along with theoretically relevant variables were then included in multivariable logistic regression analyses. Logistic regressions were used to estimate odds ratios for predictors of vaccination while adjusting for relevant covariates. Model fit was assessed using the Hosmer and Lem tests. A priori variables were included based on psychosocial constructs from the questionnaire hypothesized to influence vaccination rates. Statistically significance was set at *p* < 0.05.

## Results

### Demographics

Four hundred participants consented; 200 were randomized to the intervention group and 200 to the control group. Participant demographics are shown in Table 1; groups did not differ significantly. Retention between years 1 and 2 was high. In the control group, 192 participants (96.0%) completed both phases of the study; 6 were lost to follow-up, 1 died, and 1 refused vaccination. In the intervention group, 183 participants (91.5%) were retained; 14 were lost to follow-up, 2 died, and 1 patient was incarcerated. All patients in the intervention arm received the vaccine education sessions and a reminder to be immunized during the visit. The standard vaccine order set was part of the medical record and available to both groups. In the control group, fewer than 10% attended regular education classes.

**Table 1:**
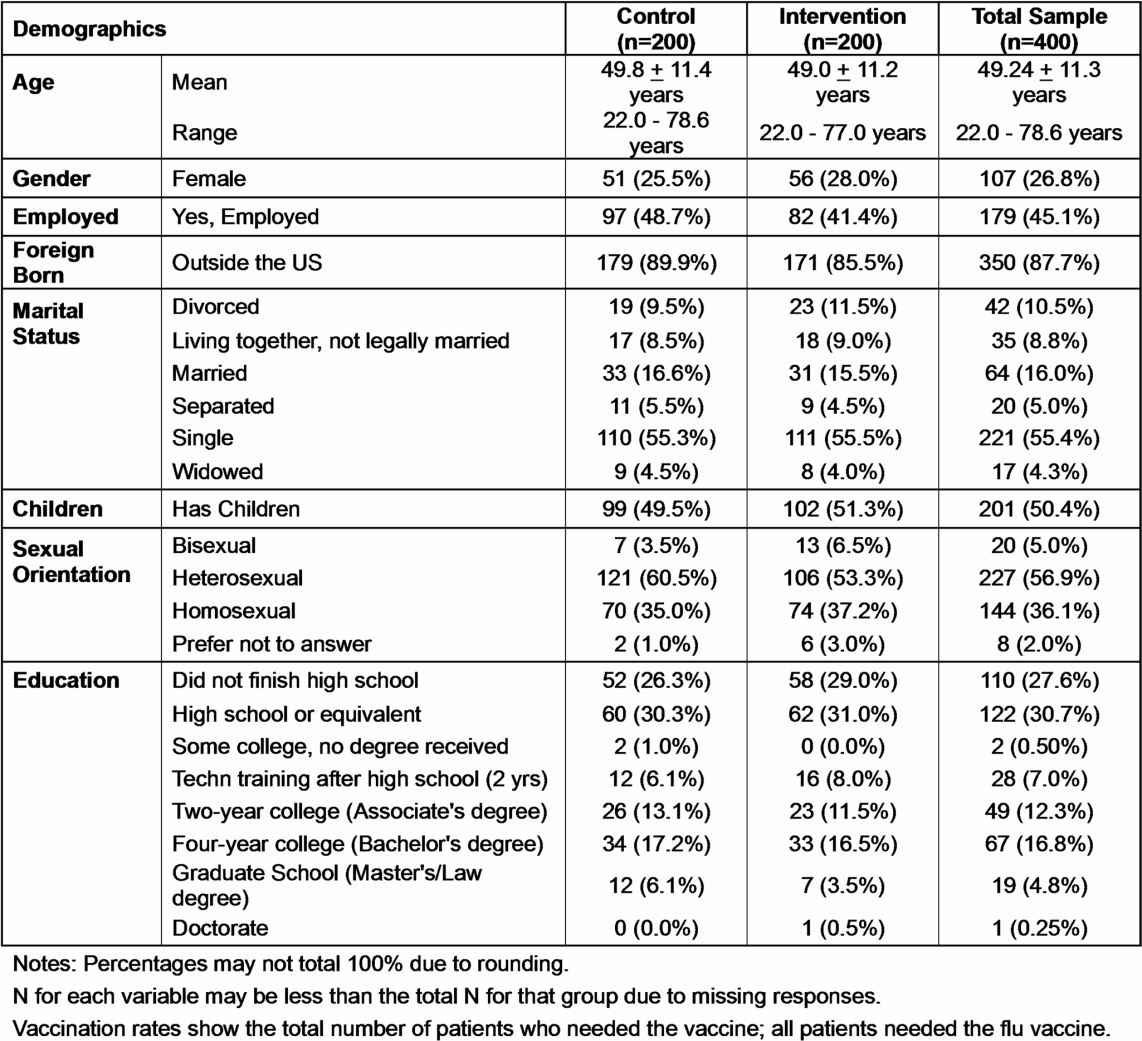
Demographics and immunizations by group

### Vaccination Rates

There were no significant differences between groups in influenza or pneumococcal vaccination rates. No between-group differences were found in either study year; all chi-square tests yielded *p* > 0.05. Vaccination rates are presented in Table 2.

**Table 2:** Vaccination rates UM/JMH HIV outpatient clinic: baseline, national averages and end of 1st and 2nd year

	UM/JMH baseline^a^	National average^b^	End of YR1 control	End of YR1 intervention	End of YR2 control	End of YR2 intervention
Influenza	46%	65%	61.5% (123/200)	62% (124/200)	61.9% 120/192	68% (124/183)
Pneumovax^c^	35%	70%	87%	88%	87.5%	83%
Prevnar^d^	N/A		80.5%	79%	96%	90%

^a^Internal historical data

^b^Source: HIVQual-US Annual Data Report, 2011

^c^Calculated as total number of patients with documented Pneumovax vaccine in the cohort

^d^Calculated as total number of patients with documented Prevnar vaccine in the cohort

Influenza vaccination coverage was 61.8% in 2015 and 65.0% in 2016. Pneumovax coverage was 87.5% in 2015 and 85.0% in 2016. Prevnar coverage was 79.8% in 2015 and 92.8% in 2016.

For influenza, after adjusting for age, patients vaccinated in 2015 were more than twice as likely to be vaccinated again in 2016 (OR = 2.37, 95% CI 1.54–3.63), regardless of study group.

### Vaccination Rates by Providers

Vaccination rates varied widely by provider, ranging from 25% to 88.9%. Statistical analyses were not performed because some providers had too few patients.

#### Psychosocial Factors as Predictors of Immunization

Health literacy did not differ between groups and did not independently predict immunization rates in either year. Because health literacy can influence other psychosocial factors, it was considered as a moderating variable in the subsequent analyses. Immunization rates were combined across all three vaccines within each year because not all patients required new Pneumovax or Prevnar doses. Using individual new immunization rates produced small sample sizes that did not allow meaningful logistic regression analyses with multiple predictors and covariates.

#### Psychosocial Factors as Predictors of Immunization—Medication Adherence

Vaccination rates were somewhat related to medication adherence, which included behavioral, motivational (personal and social), and information components. In year 1, medication adherence was not associated with immunization rates. In 2016, after adjusting for age and health literacy, patients with higher information skills scores were more likely to be vaccinated (OR = 1.05, 95% CI 1.00–1.11), regardless of group. The effect remained even after adjusting for previous vaccination in 2015 along with age and health literacy; regardless of intervention group, patients who had the vaccine in 2015 (OR = 2.47, 95% CI 1.60–3.81) and had higher scores on the information skills were more likely to receive a vaccine (OR = 1.05, 95% CI 1.00–1.11). Neither the behavioral nor motivational factors were significantly associated with vaccination rates in either year.

#### Psychosocial Factors as Predictors of Immunization—HIV Knowledge

HIV knowledge was not a significant predictor of immunization in either year of the study (Year 1: OR = 0.56, 95% CI  0.20–1.60; Year 2: OR = 1.47, 95% CI  0.50–4.32) even after adjusting for age, health literacy, and intervention group (Year 1: OR = 0.56, 95% CI  0.19–1.66; Year 2: OR = 1.39, 95% CI  0.49–3.90).

#### Psychosocial Factors as Predictors of Immunization—Quality of Life

Self-rated health/quality of life was not a significant predictor of immunization rates (Year 1: OR = 1.09, 95% CI  0.92–1.28; Year 2: OR = 1.13, 95% CI  0.95–1.34). After adjusting for age, health literacy, and group, quality of life remained non-significant (Year 1: OR = 1.09, 95% CI  0.93–1.30; Year 2: OR = 1.12, 95% CI  0.94–1.33).

#### Psychosocial Factors as Predictors of Immunization—Acculturation

The Hispanic domain of the acculturation scale was not related to vaccination rates in either year (Year 1: OR = 0.86, 95% CI  0.59–1.25; Year 2: OR = 1.25, 95% CI  0.85–1.82); the relationship was not significant after controlling for age, health literacy, and intervention group (Year 1: OR = 0.86, 95% CI  0.59–1.26; OR = 1.27, 95% CI  0.86–1.87). However, for the non-Hispanic domain of the acculturation scale, in Year 2, lower scores on the non-Hispanic domain, indicating less integration or acceptance of American culture, was associated with higher vaccination rates (OR = 0.74, 95% CI  0.60–0.92); this relationship remained significant after adjusting for age, health literacy, and intervention group (OR = 0.70, 95% CI  0.55–0.89). In Year 1, this relationship was not significant (OR = 0.94, 95% CI 0.81–1.22), even after adjustment (OR = 0.99, 95% CI 0.79–1.25). Acculturation also assessed language use, proficiency, and electronic media engagement; in Year 2, lower English proficiency was associated with higher vaccination rates (OR = 0.53, 95% CI  0.35–0.80); this relationship remained significant after adjusting for age, health literacy, and intervention group (OR = 0.45, 95% CI  0.28–0.72). Language use nor electronic media engagement were significantly related to vaccination rates in either year.

## Discussion

Vaccination rates in this clinic increased after the intervention, compared to its historic rates and approached or exceeded national rates in similar settings. However, rates were similar in both groups, so the intervention’s individual-level effect cannot be confirmed. One explanation is that control patients benefited indirectly from intervention activities, as provider reminders and heightened clinic-wide awareness emphasized vaccination for all patients. Control patients may also have learned about vaccination benefits through interactions with intervention patients in waiting rooms or through general clinic activities such as standard education sessions. Patients with personal or family ties may have shared vaccination information with one another. This cross-group likely reduced differences between the control and intervention groups but had an overall positive impact on clinic-wide vaccination rates. General increases in public awareness (e.g., media campaigns) may also have influenced vaccination uptake across both groups after study initiation. However, that was only observed after the study was implemented. Despite no significant group or year differences, study participants had higher vaccination rates compared to the rate on our clinic population the year prior to the intervention (46% for influenza vaccine) and similar to immunization rates in similar cohorts in the US and abroad. For example, *HIVqual* reported that 65% influenza vaccination coverage. A 2015 report from the HIV Outpatient Study (HOPS) found annual vaccination rates ranging from 26.4% to 50.9% [32], described annual vaccination rates that ranged from 26.4% to 50.9%. A Danish survey of 203 PLWH reported uptake of 31% for influenza and 4.4% for pneumococcal vaccines [33]. A survey of PLWH across 16 hospitals in Paris found 61.2% pneumococcal and 44.7% influenza coverage [34]. For pneumococcal vaccination, the national average was 70% (HIVQual-US, HIVQual-US Annual Data Report, 2011), while the historic average in our clinic rate was only 35% compared to over 80% during the study.

Another key finding was that influenza vaccination in year 1 strongly predicted vaccination in year 2. This suggests vaccination behaviors may have been established early in care, regardless of intervention group.

We also observed wide variation in vaccination rates of patients according to their medical providers. This was an intriguing finding although a strong statistical association was not found because there was also a large range of patients that each provider had in the study, ranging from 2 to 100. It is unclear the reasons of this large variation in vaccination rates according to providers. However, understanding that as one of the most frequent reasons for non-vaccination is that of not being suggested by the physician [34] this should be further explored. Potential factors include the provider’s own perceptions of the importance of vaccines, other various factors, such as clinic scheduling and study staff availability. It may be important to pursue additional research to understand the factors which may contribute to variance in vaccination rates between providers.

Although lack of physician recommendation is a frequent reason for non-vaccination [35], ultimately, patient agreement to receive vaccines remains a personal decision. With the instruments used in this study, we were unable to find motivation or behavioral factors that could explain this different preference for vaccination. Health literacy alone was not related to vaccination rates; these behavioral analyses were performed across all patients and between intervention/control groups. An intriguing finding was that in this patient population, lower integration of US culture and less English language proficiency was associated with better immunization rates. This underscores the importance of culturally tailored interventions in this population and suggests avenues to improve vaccine uptake.

At an individual level there was no difference in the impact of our intervention in vaccination rates. However, when looking at the pre and post intervention period it is strongly suggested that a clinic wide intervention can increase uptake of recommended vaccinations in inner-city clinics that serve patients with HIV infection. Clinics with below-average vaccination rates may rapidly improve to national standards. Additional studies exploring the reasons of no-vaccination even when clinic wide interventions are implemented are needed to better understand barriers and facilitators of these interventions and should be centered around providers, patients, and clinic flow. Future research should further explore how attitudes toward vaccination differ among providers whose patients have high and low vaccination rates. In summary, this study adds to the understanding of effective strategies to increase vaccination uptake in urban HIV care settings and highlights the need for further research into provider and patient-centered barriers and facilitators.
